# *Trans*-Acting Effectors Versus RNA *Cis*-Elements: A Tightly Knit Regulatory Mesh

**DOI:** 10.3389/fmicb.2020.609237

**Published:** 2020-12-15

**Authors:** Marie-Claude Carrier, Evelyne Ng Kwan Lim, Gabriel Jeannotte, Eric Massé

**Affiliations:** Department of Biochemistry and Functional Genomics, RNA Group, Université de Sherbrooke, Sherbrooke, QC, Canada

**Keywords:** translational regulation, translational determinants, *cis* regulatory elements, small regulatory RNAs, regulatory mechanisms, translational enhancers

## Abstract

Prokaryotic organisms often react instantly to environmental variations to ensure their survival. They can achieve this by rapidly and specifically modulating translation, the critical step of protein synthesis. The translation machinery responds to an array of *cis*-acting elements, located on the RNA transcript, which dictate the fate of mRNAs. These *cis*-encoded elements, such as RNA structures or sequence motifs, interact with a variety of regulators, among them small regulatory RNAs. These small regulatory RNAs (sRNAs) are especially effective at modulating translation initiation through their interaction with *cis*-encoded mRNA elements. Here, through selected examples of canonical and non-canonical regulatory events, we demonstrate the intimate connection between mRNA *cis*-encoded features and sRNA-dependent translation regulation. We also address how sRNA-based mechanistic studies can drive the discovery of new roles for *cis*-elements. Finally, we briefly overview the challenges of using translation regulation by synthetic regulators as a tool.

## Introduction

**FIGURE 1:**
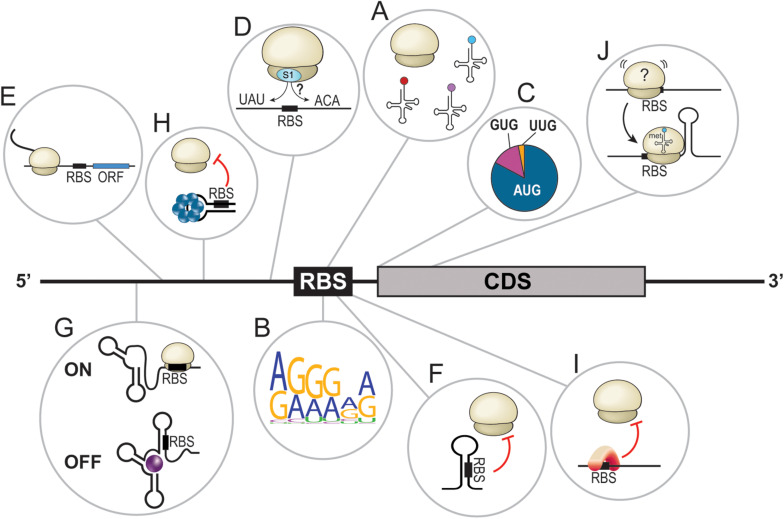
Overview of bacterial translational determinants. **(A)** Proper assembly of the translational machinery, including the ribosomes and tRNAs, is required for efficient translation. **(B)** Graphical representation (Crooks et al., 2004) of the Shine-Dalgarno core sequence in *E. coli* based on 50 random mRNAs. **(C)** Distribution of the different initiator codons in bacteria. AUG = 80%, GUG = 12%, and UUG = 8%. **(D)** Translation enhancers are often A/U- or C/A-rich sequences located upstream or downstream of the ribosome binding site (RBS). **(E)** Leader open reading frames dictate the translation of downstream CDS. **(F)** Secondary structures in the 5′UTR sequester the RBS and hinder translation by steric inhibition. **(G)** Riboswitches are complex RNA structures in 5′UTRs that, upon binding of a ligand, change conformation to turn the translation of the downstream open reading frame ON or OFF. **(H)** The binding of a protein forces the adoption of a 5′UTR structure that sequesters the RBS, preventing translation initiation. **(I)** Binding of a protein to the RBS prevents translation initiation. **(J)** A stem loop at the beginning of the coding sequence seems to stabilize the ribosome at the RBS and facilitate translation initiation.

Prokaryotic organisms depend on protein synthesis to grow and adjust to their surroundings. The inability to produce functional gene products could result in bacterial cell growth inhibition. At the forefront of protein synthesis is the translational machinery, which requires the following elements: an mRNA, the 30S ribosomal subunit (small), three initiation factors (IF1, IF2, and IF3), an initiator tRNA, and the 50S ribosomal subunit (large). Together, these elements form the 70S translation initiation complex (Laursen et al., 2005; Gualerzi and Pon, 2015). Whether they are proteins or RNA molecules, translational machinery components must be synthesized, assembled properly, and available (Figure 1A). In eubacteria, most ribosomal RNAs (rRNAs) are encoded in polycistronic transcripts that must be precisely processed through multistep pathways to be functional (Deutscher, 2009). The presence of mutations in rRNAs or defect in their processing, causing mis-assembly of ribosomal subunits, can lead to rapid rRNA degradation (Deutscher, 2009; Basturea et al., 2011). The same type of quality control is also applied to both synthesis and maturation of tRNAs (Shepherd and Ibba, 2015). Furthermore, translation initiation often requires formylation of the initiator tRNA^Met^ by the methionyl-tRNAfMet transformylase (FMT) as disruption of the *fmt* gene leads to important growth defects (Guillon et al., 1992).

Translational regulation through modulation of its machinery affects protein synthesis at a cellular scale. Although very effective, this is of little help when regulation of specific genes is required. To palliate this, prokaryotic organisms have developed an array of *cis*- and *trans*-acting strategies responding to environmental and cellular cues modulating translation of specific mRNAs.

The first *cis*-acting regulator of translation is the sequence of the mRNA itself, especially the translation initiation region (TIR; Osterman et al., 2013). Examples of TIR features include, among others: (I) the Shine-Dalgarno sequence (SD); (II) the initiation codon, with either the canonical AUG or alternative codons such as GUG or UUG (Villegas and Kropinski, 2008); (III) translational enhancers (TEs) such as A/U and C/A-rich sequences; and (IV) leader open reading frames (Figures 1B–E; de Smit and van Duin, 2003; Andreeva et al., 2018; Sterk et al., 2018; Romilly et al., 2019). Dictated by the primary sequence, the mRNA structure is also a major *cis*-acting regulator of translation. Simple structures, such as stem loops, prevent translation initiation through the sequestration of crucial ribosome binding site (RBS) elements, especially the SD (Figure 1F). More complex structures, called riboswitches, can also be found in 5′ untranslated regions (UTRs) of certain mRNAs. Riboswitches respond to the presence of specific molecules called ligands (e.g., metabolites, vitamins, coenzymes, ions, and uncharged tRNAs, etc.). In these cases, the interaction of a riboswitch to its ligand induces conformational changes that can turn translation ON or OFF, dictating the fate of the cognate mRNA (Figure 1G; Abduljalil, 2018; Pavlova et al., 2019; Bédard et al., 2020). Moreover, biochemical factors modulating the structure modification of riboswitches include temperature (thermosensors; Schumann, 2012; Loh et al., 2018; Mandin and Johansson, 2020) or pH (Nechooshtan et al., 2009).

*Trans*-acting translational regulators also play lead roles in dictating and redirecting gene expression. RNA-binding proteins (RBPs) are known to interact with various *cis*-elements to alter the secondary structure of an mRNA (Figure 1H) or to directly interfere with translation initiation (Figure 1I). This short review will focus on ribonucleic *trans*-acting regulators, particularly small regulatory RNAs (sRNAs), interacting with mRNA *cis*-elements to modulate their translation. Even though certain mechanisms of action are common and well described, we will overview how discovery of novel canonical regulatory events is still critical in our understanding of how bacteria adapt to their environment. Then, uncommon sRNA-dependent regulatory mechanisms targeting *cis*-elements will also be explored. Finally, a brief outlook on the synthetic use of *cis*-dependent translation regulation will be provided.

## Bacterial Non-Coding Regulatory RNAs

### What Are *Trans*-Acting Small Regulatory RNAs

Bacterial *trans*-acting small regulatory RNAs are powerful regulators of gene expression. Acting through a tight network of regulation, sRNAs are responsible for the maintenance of cellular homeostasis and virulence. Their synthesis quickly responds to environmental signals, making them efficient stress-response regulators. Typically, sRNAs base-pair to their target mRNAs to repress or increase protein synthesis through various mechanisms of action, most of which have been extensively reviewed elsewhere (Gottesman and Storz, 2011; Papenfort and Vanderpool, 2015; Carrier et al., 2018; Dutta and Srivastava, 2018). These regulatory events result in modulation of mRNA stability and/or of translation efficiency. Even though sRNAs have been studied for decades now, identification of new sRNAs and sRNA targets helps understand how bacterial cells adapt to their environment. Moreover, additional regulatory mechanisms are periodically brought up to light, creating a more accurate portrait of sRNA complexity.

### Canonical Mechanisms of Action Are Still up to Date

One of the most characterized mechanisms employed by sRNAs is the targeting of the RBS to hinder translation initiation. New examples of sRNAs using this mechanism are still discovered to this day. In *Salmonella enterica* serovar Typhimurium, the PinT sRNA has recently been found to regulate two mRNAs, *rtsA* and *hilA*, through a canonical mechanism (Kim et al., 2019). PinT pairs to the 5′UTR of both mRNAs, near the SD of *rtsA* and near the start codon of *hilA*. In both cases, the interaction blocks translation initiation by preventing ribosome assembly at the RBS. While PinT interaction is enough to repress *hilA* mRNA, the activity of the ribonuclease E (RNase E) is required to fully repress *rtsA* (Kim et al., 2019). The identification of these new mRNA targets regulated by the PinT sRNA added new hindsight on *Salmonella* transition from the invasion stage to intracellular growth during infection.

Through a wide range of mechanisms, sRNAs can also positively regulate mRNA targets. The most common of those mechanisms involves the pairing of an sRNA to the 5′UTR of an mRNA, upstream from the TIR. This causes structural modifications facilitating ribosome assembly and translation initiation. Notably, sRNA-based regulation has evolved to accommodate peculiarities of different mRNAs, resulting in many variations of canonical regulatory mechanisms. For instance, in the enterohemorrhagic *Escherichia coli* (EHEC), the *pchA* mRNA, encoding a transcriptional activator, is regulated in *cis* by secondary structures in its own transcript. Indeed, the *pchA* mRNA coding sequence (CDS) presents an anti-Shine-Dalgarno (anti-SD) sequence, which forces the folding of the mRNA on itself through strong interactions with the SD. The resulting double-stranded RNA structure sequesters the RBS of *pchA*, thus inhibiting translation initiation (Melson and Kendall, 2019). This structure, however, is sensitive to a *trans*-regulator, the sRNA DicF. It has recently been found to pair within the CDS of *pchA*, at the anti-SD, to prevent the self-folding of *pchA*. This sRNA:mRNA interaction results in facilitated translation initiation by rendering the RBS of *pchA* accessible to ribosomes. Data indicate that the presence of DicF paired in the CDS of *pchA* does not impair translation elongation rates, suggesting that elongating ribosomes are able to displace the sRNA from the mRNA.

What is the cellular advantage of an mRNA being regulated in such a context? Translation inhibition often leads to mRNA degradation since it is not protected by translating ribosomes. However, the *pchA* mRNA does not seem to be destabilized when translation is OFF compared to a mutated version of *pchA* unable to form the anti-SD:SD interaction (translation ON; Melson and Kendall, 2019). Perhaps the folded structure allows translation inhibition while also protecting the transcript against degradation? If so, following DicF expression, the stable *pchA* mRNA could readily be translated, possibly allowing a rapid response to changes in environmental conditions.

The pairing of sRNAs in the CDS, outside of the five-codon window (Bouvier et al., 2008), is mostly known to cause destabilization of the target mRNA and lead to repression of gene expression (Pfeiffer et al., 2009; Fröhlich et al., 2012; Lalaouna et al., 2015b). The regulation of *pchA* by DicF is rather uncommon as the pairing of the sRNA downstream of the five-codon window directly impacts translation initiation. Moreover, another uncommon characteristic of this interaction is the fact that DicF positively regulates *pchA*, contrary to most of the regulatory events involving an sRNA pairing in the CDS of its target, demonstrating the versatility of sRNA mechanisms of actions.

### sRNAs Get Fancy: Uncommon Targeting of an mRNA Element

The SD sequence and the initiator codon are critical determinants of translation. However, other *cis*-encoded features such as TEs can play a major role in dictating translation rates. Described as A/U- or C/A-rich sequences, TEs were found, among others, in the *E. coli rnd* and *fepB* mRNAs that present the alternative initiator codons UUG and GUG, respectively (Zhang and Deutscher, 1992; Hook-Barnard et al., 2007), or in the *tuf* mRNA of *Mycoplasma genitalium*, which lacks an SD sequence (Loechel et al., 1991). TEs were originally believed to facilitate translation initiation of mRNAs presenting suboptimal SD-AUG contexts. Surprisingly, TEs were later noticed in mRNAs with optimal RBS characteristics, for example, in the *dppA* mRNA of *E. coli* and *Salmonella* (Yang et al., 2014). This suggested that optimal TIR features are not necessarily sufficient to ensure required translation initiation rates. It has been proposed that A/U- and C/A-rich sequences, such as TEs, could act as binding sites for the S1 ribosomal protein, which helps position the ribosome during translation initiation (Hauryliuk and Ehrenberg, 2006; Studer and Joseph, 2006). Considering that sRNAs are known to act through non-canonical mechanisms of action, it is not surprising that they target TEs (Sharma et al., 2007). Although not the only one, GcvB is a perfect example of a TE-targeting sRNA. GcvB negatively regulates the *dppA* mRNA by targeting a C/A-rich stretch located immediately upstream of the SD (Yang et al., 2014). It also regulates the *gltI* mRNA by pairing to a TE located 40 nucleotides (nts) upstream of the RBS (Sharma et al., 2007).

**FIGURE 2:**
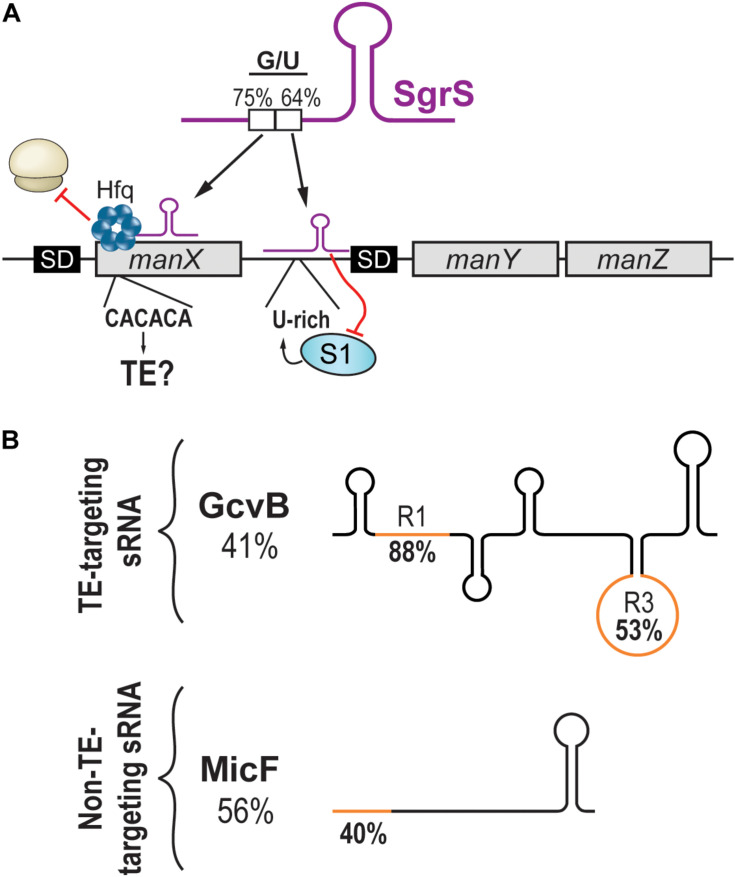
Targeting of translational enhancers by sRNAs. **(A)** The *manXYZ* polycistronic transcript is dually regulated by two regions of the sRNA SgrS. SgrS interacts with the coding sequence of *manX* and recruits Hfq to block translation. The pairing site on *manX* presents a C/A-rich sequence that could be a translational enhancer. On the other hand, SgrS interacts with the 5′UTR of *manY* and masks a U-rich translational enhancer, hindering the translation of *manY* and *manZ*. **(B)** Schematic representation of sRNAs targeting translational enhancers (upper panel) or not (lower panel). Percentage of G/U of the full sRNA sequence is indicated under the sRNA name. The G/U content of the seed region (orange) is indicated on the sRNA drawing.

While studying the regulation of the *manXYZ* polycistronic transcript by the SgrS sRNA, the Vanderpool lab discovered that SgrS targets *manXYZ* at two distinct base-pairing sites, exerting its regulatory effect through two different mechanisms of action (Figure 2A). First, SgrS pairs in the CDS of *manX*, recruiting the RNA chaperone Hfq to repress *manX* synthesis (Rice and Vanderpool, 2011; Azam and Vanderpool, 2018). Second, using a different sequence, SgrS pairs 30 nts upstream of *manY* SD and dictates the fate of both *manY* and *manZ*. It was elegantly demonstrated that the *manY* region targeted by SgrS is a U-rich TE actively participating in the translation of *manY* through its interaction with the S1 ribosomal protein (Azam and Vanderpool, 2020). The working model of this regulation suggests that the S1:mRNA complex remodels the SD, favoring translation. SgrS prevents this activation by masking the TE site and impairing translation initiation.

Whereas the repression of *manY* occurs through the occlusion of a U-rich TE, the regulation of *manX* has not been shown to involve a TE. However, the high G/U content of the SgrS region interacting with the 5′UTR of *manX* might suggest otherwise. We wondered if the target site on *manX* presented features of a C/A-rich TE. A stretch of C/A-rich (CACACA) sequence was indeed found, suggesting that translation initiation of *manX* could depend on the S1 protein, which seems to favor A/U- and C/A-rich sequences (Figure 2A). If demonstrated, this would indicate that SgrS action on *manX* involves more than the recruitment of Hfq to achieve translation inhibition.

Like SgrS, which uses two different pairing sites, the sRNA GcvB possesses two distinct seed regions called R1 and R3. The regulation of both *dppA* and *gltI* mRNAs involves R1, which presents a high G/U content (88%), indicating its potential at targeting C/A-rich regions. In contrast to this, the R3 seed region of GcvB has a lower G/U content (53%), and its targets show little potential at encoding C/A-rich TEs (Figure 2B; Lalaouna et al., 2019). Therefore, would it be possible to predict an sRNA targeting C/A-rich TEs from its G/U content?

Analysis of the MicF sRNA, which is not known to target TEs, exposed a possible barrier to such predictions. Indeed, the entire sequence of MicF presents a moderate G/U content (56%), suggesting some potential at targeting TEs. However, MicF seed region (nucleotides 1–15) presents a very low G/U content (40%), indicating that actual chances of targeting C/A-rich TEs are modest (Figure 2B). This remarkable difference between whole sequence versus seed region G/U content is also observed with GcvB. The R1 seed region of GcvB has a very high G/U content (88%) compared to an overall very low G/U content (41%; Figure 2B). The question is, how would predictions be achieved for sRNAs with no obvious seed regions such as RyhB? In addition to the sequence of the binding region, would other sRNA and/or mRNA features be required for this mechanism of action to occur? Are TEs so versatile in their sequences that no prediction can be performed?

### sRNAs Studies Help Redefine Roles of Translational Determinants

While investigating the targetome of both OmrA and OmrB sRNAs (hereby referred to as OmrA/B), Guillier and colleagues identified a negatively regulated target, the *fepA* mRNA (Jagodnik et al., 2017). They show that OmrA/B represses *fepA* by hindering translation initiation. Interestingly, both sRNAs interact with nucleotides downstream of the five-codon window usually targeted by repressing sRNAs (Bouvier et al., 2008). In this case, OmrA/B represses translation by disrupting a stem loop (SL) in the CDS of *fepA*, suggesting that the SL itself might favor translation. This is in direct contrast with previous observations concerning SLs. When located in 5′UTRs, SLs hinder the recognition of TIR elements through a sequestration mechanism. In many cases, this inhibition is alleviated with the help of sRNAs interacting with the SL, forcing it to open. Examples include *E. coli* ArcZ, DsrA, and RprA sRNAs, all increasing the translation of *rpoS* (Kim and Lee, 2020). Another example is the sRNA RNAIII, which disrupts *hla* 5′UTR inhibitory SL to increase α-hemolysin synthesis in *Staphylococcus aureus* (Morfeldt et al., 1995). When located in the CDS, SLs are believed to slow down the elongation rate of translating ribosomes and are known to induce frameshifts (Kim et al., 2014). Based on their observation of OmrA/B, Guillier’s group brings the novel idea that the SL structure favors translation of *fepA* CDS at an early stage of initiation (Jagodnik et al., 2017). They hypothesized a mechanism of activation in which the CDS-located SL acts as a starting block to help properly position the 30S ribosomal subunit and favor the formation of the translation initiation complex (Figure 1J). A similar activator SL, also targeted by OmrA/B, was found in the *bamA* mRNA (Jagodnik et al., 2017).

### What Are Bacterial sRNAs Not Doing…Yet?

Bacterial sRNAs act through plentiful regulatory mechanisms and target different types of RNA molecules such as mRNAs or even other sRNAs (Figueroa-Bossi et al., 2009; Miyakoshi et al., 2015; Lalaouna et al., 2015a). Their impact on genomic expression and the resulting physiological effects have been extensively studied. However, to our knowledge, the core of the translation machinery, i.e., the ribosomes, is not directly targeted by sRNAs in prokaryotes. This contrasts with eukaryotic and archaeal organisms, in which direct association of non-coding RNAs to the translational apparatus has been shown. In 2012, Gebetsberger et al. (2012) demonstrated that in the archaea *Haloferax volcanii*, the association of a tRNA-derived fragment (tRF) to the 30S ribosomal subunit globally downregulates translation in conditions of hyperosmotic stress. In similar stressful conditions, the association of a *Saccharomyces cerevisiae* exon-derived RNA to the 60S ribosomal subunit can hinder translation *in vitro* (Pircher et al., 2014). More recently, a tRF in *Trypanosoma brucei* has been shown to promote protein synthesis through its direct association with the translational machinery (Fricker et al., 2019). Examples above involve RNA fragments that are relatively short (less than 50 nts) compared to the canonical bacterial sRNAs, averaging 100 nts in length. Bacterial cells, however, are not devoid of extremely short, stable RNA molecules. Many tRFs have been identified in bacteria; however, their functions remain mostly unexplored. Could their investigation reveal that, just as in eukaryotes and archaea, these short non-coding RNAs can find their way into the bacterial translational machinery?

The question of why bacterial regulatory RNAs have not yet been found to target rRNAs is still in suspense. A possible explanation could be related to experimental procedures rather than experimental limitations. Most high-throughput experiments heavily rely on the depletion of rRNAs prior to sequencing to produce analyzable data. Indeed, rRNAs are so abundant that their depletion becomes necessary to obtain enough reads from other RNAs (e.g., mRNAs, tRNAs, and sRNAs; Yang et al., 2011). However, rRNA removal also creates a bias, preventing the identification of sRNA:rRNA interactions. Future breakthroughs in RNA sequencing techniques might resolve this bias and allow the identification of a new class of small regulatory RNAs that directly target the translational machinery. Moreover, optimized techniques could lead to the identification of novel, conserved non-coding RNAs, such as those produced by pervasive transcription (Lybecker et al., 2014). For example, these transcripts could act as asRNAs, regulating components of the translational machinery.

From there, many questions might arise. Would sRNAs target all ribosomes with no selection or would they interact solely with specific specialized ribosomes? The current understanding of bacterial specialized ribosomes is still limited. These ribosomes could be generated via a modification in component stoichiometry (Chen et al., 2020) or include/exclude ribosomal proteins, such as the SRA protein (van de Waterbeemd et al., 2017). In turn, these modifications could modify the accessible parts of the rRNA, exposing novel base-pairing sites for sRNAs. However, would the base pairing of the sRNA to the rRNA be the sole determinant of the interaction? Examples of sRNAs interacting with proteins are numerous. To name only a few, their interaction with chaperone proteins such as Hfq and ProQ (Smirnov et al., 2016; Kavita et al., 2018) or with regulatory proteins such as CsrA (Liu et al., 1997; Weilbacher et al., 2003) is proof that sRNAs have protein-binding properties. Therefore, one could hypothesize that sRNAs targeting ribosomes could do so through interaction with ribosomal proteins.

## Outlooks: Using *Trans*-Regulation of *Cis*-Elements as a Tool

Regulation of translation is a tightly controlled process essential to bacterial survival and fitness. Therefore, it offers a great opportunity to use translation regulation as a tool for diverse applications.

Currently, the massive use of antibiotics in both clinical and agricultural settings fuels the emergence of drug-resistant bacterial strains. Moreover, with the rising importance of microbiomes as beneficial health factors, the use of large spectrum antibiotics to fight bacterial infections does not appear suitable for chronic treatments as it leads to microbiome dysbiosis (Panda et al., 2014; Becattini et al., 2016), urging the scientific community to develop new antimicrobial drugs or identify new drug targets (Ventola, 2015). Unsurprisingly, a great number of antimicrobial compounds, whether they are from natural or synthetic origin, target translation (Sohmen et al., 2009; Nikolay et al., 2016; Champney, 2020). An innovative approach, termed antisense therapy, uses artificial antisense oligonucleotides (ASOs) to repress the translation of single mRNAs through base-pairing complementarity (Dias and Stein, 2002). ASO therapy strategies involve targeting antibiotic resistance genes (Daly et al., 2017; Sully et al., 2017; Kauss et al., 2020) or essential genes (Sawyer et al., 2013). It is tempting to assume that designing ASOs targeting the TIR of an mRNA would be specific enough to prevent off-target regulation. However, these elements are somewhat conserved between mRNAs and, more importantly, are conserved in closely related species. Since it is clear that inhibition of translation can occur through the targeting of *cis*-elements outside of the RBS by bacterial sRNAs, ASOs are now being developed to target these more complex and less conserved sequences. Even though this vision of ASOs as antibiotics is still facing major hurdles, technological breakthroughs bring us closer to this achievement every year [for a review, see Vogel (2020)].

## Concluding Remarks

Translational regulation is a layered process depending on the translational machinery and on a variety of elements encoded in *cis* or in *trans* (Figure 1). *Trans*-acting regulators, especially sRNAs, have evolved to target *cis*-elements, some more commonly (e.g., SD, RBS) than others (e.g., translational enhancers). Thanks to rapidly evolving high-throughput RNA sequencing studies, new regulatory events are periodically brought up to light. While identification of new sRNA targets regulated through canonical mechanisms of action mainly helps to understand cellular physiology and bacterial adaptation to its environment, non-canonical events can lead to much more. Indeed, identification of new mechanisms of sRNA-dependent regulation is crucial to expand the boundaries of current regulatory networks. Moreover, in some instances, studies based on unusual regulatory events contribute to the identification of novel roles for *cis*-elements, strengthening the importance of studying sRNA mechanisms.

## Author Contributions

M-CC, ENKL, and GJ wrote the manuscript. M-CC prepared the figures. EM revised the manuscript. All authors contributed to the article and approved the submitted version.

## Conflict of Interest

The authors declare that the research was conducted in the absence of any commercial or financial relationships that could be construed as a potential conflict of interest.

## References

[B1] Abduljalil J. M. (2018). Bacterial riboswitches and RNA thermometers: nature and contributions to pathogenesis. *Noncoding RNA Res.* 3 54–63. 10.1016/j.ncrna.2018.04.003 30159440 PMC6096418

[B2] Andreeva I. Belardinelli R. Rodnina M. V. (2018). Translation initiation in bacterial polysomes through ribosome loading on a standby site on a highly translated mRNA. *PNAS* 115 4411–4416. 10.1073/pnas.1718029115 29632209 PMC5924895

[B3] Azam M. S. Vanderpool C. K. (2018). Translational regulation by bacterial small RNAs via an unusual Hfq-dependent mechanism. *Nucleic Acids Res.* 46 2585–2599. 10.1093/nar/gkx1286 29294046 PMC5861419

[B4] Azam M. S. Vanderpool C. K. (2020). Translation inhibition from a distance: the small RNA SgrS silences a ribosomal protein S1-dependent enhancer. *Mol. Microbiol.* 114 391–408. 10.1111/mmi.14514 32291821 PMC7502529

[B5] Basturea G. N. Zundel M. A. Deutscher M. P. (2011). Degradation of ribosomal RNA during starvation: comparison to quality control during steady-state growth and a role for RNase PH. *RNA* 17 338–345. 10.1261/rna.2448911 21135037 PMC3022282

[B6] Becattini S. Taur Y. Pamer E. G. (2016). Antibiotic-induced changes in the intestinal microbiota and disease. *Trends Mol. Med.* 22 458–478. 10.1016/j.molmed.2016.04.003 27178527 PMC4885777

[B7] Bédard A.-S. V. Hien E. D. M. Lafontaine D. A. (2020). Riboswitch regulation mechanisms: RNA, metabolites and regulatory proteins. *Biochim. Biophys. Acta* 1863:194501. 10.1016/j.bbagrm.2020.194501 32036061

[B8] Bouvier M. Sharma C. M. Mika F. Nierhaus K. H. Vogel J. (2008). Small RNA binding to 5′ mRNA coding region inhibits translational initiation. *Mol. Cell* 32 827–837. 10.1016/j.molcel.2008.10.027 19111662

[B9] Carrier M.-C. Lalaouna D. Massé E. (2018). Broadening the definition of bacterial small RNAs: characteristics and mechanisms of action. *Annu. Rev. Microbiol.* 72 141–161. 10.1146/annurev-micro-090817-0 30200848

[B10] Champney W. S. (2020). Antibiotics targeting bacterial ribosomal subunit biogenesis. *J. Antimicrob. Chemother.* 75 787–806. 10.1093/jac/dkz544 31942624 PMC8453389

[B11] Chen Y.-X. Xu Z. Ge X. Hong J.-Y. Sanyal S. Lu Z. J. (2020). Selective translation by alternative bacterial ribosomes. *PNAS* 117 19487–19496. 10.1073/pnas.2009607117 32723820 PMC7431078

[B12] Crooks G. E. Hon G. Chandonia J.-M. Brenner S. E. (2004). WebLogo: a sequence logo generator. *Genome Res.* 14 1188–1190. 10.1101/gr.849004 15173120 PMC419797

[B13] Daly S. M. Sturge C. R. Felder-Scott C. F. Geller B. L. Greenberg D. E. (2017). MCR-1 inhibition with peptide-conjugated phosphorodiamidate morpholino oligomers restores sensitivity to polymyxin in *Escherichia coli*. *mBio* 8:e1315-17. 10.1128/mBio.01315-17 29114023 PMC5676038

[B14] de Smit M. H. van Duin J. (2003). Translational standby sites: how ribosomes may deal with the rapid folding kinetics of mRNA. *J. Mol. Biol.* 331 737–743. 10.1016/s0022-2836(03)00809-x 12909006

[B15] Deutscher M. P. (2009). Maturation and degradation of ribosomal RNA in Bacteria. *Prog. Mol. Biol. Transl. Sci.* 85 369–391. 10.1016/S0079-6603(08)00809-X 19215777

[B16] Dias N. Stein C. A. (2002). Antisense oligonucleotides: basic concepts and mechanisms. *Mol. Cancer Ther.* 1 347–355. 12489851

[B17] Dutta T. Srivastava S. (2018). Small RNA-mediated regulation in bacteria: a growing palette of diverse mechanisms. *Gene* 656 60–72. 10.1016/j.gene.2018.02.068 29501814

[B18] Figueroa-Bossi N. Valentini M. Malleret L. Bossi L. (2009). Caught at its own game: regulatory small RNA inactivated by an inducible transcript mimicking its target. *Genes Dev.* 23 2004–2015. 10.1101/gad.541609 19638370 PMC2751969

[B19] Fricker R. Brogli R. Luidalepp H. Wyss L. Fasnacht M. Joss O. (2019). A tRNA half modulates translation as stress response in Trypanosoma brucei. *Nat. Commun.* 10:118. 10.1038/s41467-018-07949-6 30631057 PMC6328589

[B20] Fröhlich K. S. Papenfort K. Berger A. A. Vogel J. (2012). A conserved RpoS-dependent small RNA controls the synthesis of major porin OmpD. *Nucleic Acids Res.* 40 3623–3640. 10.1093/nar/gkr1156 22180532 PMC3333887

[B21] Gebetsberger J. Zywicki M. Künzi A. Polacek N. (2012). tRNA-derived fragments target the ribosome and function as regulatory non-coding RNA in *Haloferax volcanii*. *Archaea* 2012:e260909. 10.1155/2012/260909 23326205 PMC3544259

[B22] Gottesman S. Storz G. (2011). Bacterial small RNA regulators: versatile roles and rapidly evolving variations. *Cold Spring Harb. Perspect. Biol.* 3:a003798. 10.1101/cshperspect.a003798 20980440 PMC3225950

[B23] Gualerzi C. O. Pon C. L. (2015). Initiation of mRNA translation in bacteria: structural and dynamic aspects. *Cell. Mol. Life Sci.* 72 4341–4367. 10.1007/s00018-015-2010-3 26259514 PMC4611024

[B24] Guillon J. M. Mechulam Y. Schmitter J. M. Blanquet S. Fayat G. (1992). Disruption of the gene for Met-tRNA(fMet) formyltransferase severely impairs growth of *Escherichia coli*. *J. Bacteriol.* 174 4294–4301. 10.1128/jb.174.13.4294-4301.1992 1624424 PMC206212

[B25] Hauryliuk V. Ehrenberg M. (2006). Two-step selection of mRNAs in initiation of protein synthesis. *Mol. Cell* 22 155–156. 10.1016/j.molcel.2006.04.004 16630885

[B26] Hook-Barnard I. G. Brickman T. J. McIntosh M. A. (2007). Identification of an AU-rich translational enhancer within the *Escherichia coli* fepB leader RNA. *J. Bacteriol.* 189 4028–4037. 10.1128/JB.01924-06 17400738 PMC1913407

[B27] Jagodnik J. Chiaruttini C. Guillier M. (2017). Stem-loop structures within mRNA coding sequences activate translation initiation and mediate control by small regulatory RNAs. *Mol. Cell* 68 158.e3–170.e3. 10.1016/j.molcel.2017.08.015 28918899

[B28] Kauss T. Arpin C. Bientz L. Vinh Nguyen P. Vialet B. Benizri S. (2020). Lipid oligonucleotides as a new strategy for tackling the antibiotic resistance. *Sci. Rep.* 10:1054. 10.1038/s41598-020-58047-x 31974472 PMC6978458

[B29] Kavita K. de Mets F. Gottesman S. (2018). New aspects of RNA-based regulation by Hfq and its partner sRNAs. *Curr. Opin. Microbiol.* 42 53–61. 10.1016/j.mib.2017.10.014 29125938 PMC10367044

[B30] Kim H.-K. Liu F. Fei J. Bustamante C. Gonzalez R. L. Tinoco I. (2014). A frameshifting stimulatory stem loop destabilizes the hybrid state and impedes ribosomal translocation. *PNAS* 111 5538–5543. 10.1073/pnas.1403457111 24706807 PMC3992627

[B31] Kim K. Palmer A. D. Vanderpool C. K. Slauch J. M. (2019). The small RNA PinT contributes to PhoP-mediated regulation of the *Salmonella* pathogenicity island 1 type III secretion system in *Salmonella enterica* Serovar Typhimurium. *J. Bacteriol.* 201 e312–e319. 10.1128/JB.00312-19 31262841 PMC6755756

[B32] Kim W. Lee Y. (2020). Mechanism for coordinate regulation of rpoS by sRNA-sRNA interaction in *Escherichia coli*. *RNA Biol.* 17 176–187. 10.1080/15476286.2019.1672514 31552789 PMC6973317

[B33] Lalaouna D. Carrier M.-C. Semsey S. Brouard J.-S. Wang J. Wade J. T. (2015a). A 3’ external transcribed spacer in a tRNA transcript acts as a sponge for small RNAs to prevent transcriptional noise. *Mol. Cell* 58 393–405. 10.1016/j.molcel.2015.03.013 25891076

[B34] Lalaouna D. Morissette A. Carrier M.-C. Massé E. (2015b). DsrA regulatory RNA represses both hns and rbsD mRNAs through distinct mechanisms in *Escherichia coli*. *Mol. Microbiol.* 98 357–369. 10.1111/mmi.13129 26175201

[B35] Lalaouna D. Eyraud A. Devinck A. Prévost K. Massé E. (2019). GcvB small RNA uses two distinct seed regions to regulate an extensive targetome. *Mol. Microbiol.* 111 473–486. 10.1111/mmi.14168 30447071

[B36] Laursen B. S. Sørensen H. P. Mortensen K. K. Sperling-Petersen H. U. (2005). Initiation of protein synthesis in bacteria. *Microbiol. Mol. Biol. Rev.* 69 101–123. 10.1128/MMBR.69.1.101-123.2005 15755955 PMC1082788

[B37] Liu M. Y. Gui G. Wei B. Preston J. F. Oakford L. Yüksel U. (1997). The RNA molecule CsrB binds to the global regulatory protein CsrA and antagonizes its activity in *Escherichia coli*. *J. Biol. Chem.* 272 17502–17510. 10.1074/jbc.272.28.17502 9211896

[B38] Loechel S. Inamine J. M. Hu P. C. (1991). A novel translation initiation region from Mycoplasma genitalium that functions in *Escherichia coli*. *Nucleic Acids Res* 19 6905–6911. 10.1093/nar/19.24.6905 1762919 PMC329327

[B39] Loh E. Righetti F. Eichner H. Twittenhoff C. Narberhaus F. (2018). RNA thermometers in bacterial pathogens. *Microbiol. Spectr.* 6:RWR-0012-2017. 10.1128/microbiolspec.RWR-0012-2017 29623874 PMC11633587

[B40] Lybecker M. Bilusic I. Raghavan R. (2014). Pervasive transcription: detecting functional RNAs in bacteria. *Transcription* 5:e944039. 10.4161/21541272.2014.944039 25483405 PMC4581347

[B41] Mandin P. Johansson J. (2020). Feeling the heat at the millennium: thermosensors playing with fire. *Mol. Microbiol.* 113 588–592. 10.1111/mmi.14468 31971637

[B42] Melson E. M. Kendall M. M. (2019). The sRNA DicF integrates oxygen sensing to enhance enterohemorrhagic *Escherichia coli* virulence via distinctive RNA control mechanisms. *PNAS* 116 14210–14215. 10.1073/pnas.1902725116 31235565 PMC6628830

[B43] Miyakoshi M. Chao Y. Vogel J. (2015). Cross talk between ABC transporter mRNAs via a target mRNA-derived sponge of the GcvB small RNA. *EMBO J.* 34 1478–1492. 10.15252/embj.201490546 25630703 PMC4474525

[B44] Morfeldt E. Taylor D. von Gabain A. Arvidson S. (1995). Activation of alpha-toxin translation in Staphylococcus aureus by the trans-encoded antisense RNA. *RNAIII. EMBO J.* 14 4569–4577. 10.1002/j.1460-2075.1995.tb00136.x 7556100 PMC394549

[B45] Nechooshtan G. Elgrably-Weiss M. Sheaffer A. Westhof E. Altuvia S. (2009). A pH-responsive riboregulator. *Genes Dev.* 23 2650–2662. 10.1101/gad.552209 19933154 PMC2779765

[B46] Nikolay R. Schmidt S. Schlömer R. Deuerling E. Nierhaus K. H. (2016). Ribosome assembly as antimicrobial target. *Antibiotics* 5:18. 10.3390/antibiotics5020018 27240412 PMC4929433

[B47] Osterman I. A. Evfratov S. A. Sergiev P. V. Dontsova O. A. (2013). Comparison of mRNA features affecting translation initiation and reinitiation. *Nucleic Acids Res.* 41 474–486. 10.1093/nar/gks989 23093605 PMC3592434

[B48] Panda S. Khader I. E. Casellas F. Vivancos J. L. Cors M. G. Santiago A. (2014). Short-term effect of antibiotics on human gut microbiota. *PLoS One* 9:e95476. 10.1371/journal.pone.0095476 24748167 PMC3991704

[B49] Papenfort K. Vanderpool C. K. (2015). Target activation by regulatory RNAs in bacteria. *FEMS Microbiol. Rev.* 39 362–378. 10.1093/femsre/fuv016 25934124 PMC4542691

[B50] Pavlova N. Kaloudas D. Penchovsky R. (2019). Riboswitch distribution, structure, and function in bacteria. *Gene* 708 38–48. 10.1016/j.gene.2019.05.036 31128223

[B51] Pfeiffer V. Papenfort K. Lucchini S. Hinton J. C. D. Vogel J. (2009). Coding sequence targeting by MicC RNA reveals bacterial mRNA silencing downstream of translational initiation. *Nat. Struct. Mol. Biol.* 16 840–846. 10.1038/nsmb.1631 19620966

[B52] Pircher A. Bakowska-Zywicka K. Schneider L. Zywicki M. Polacek N. (2014). An mRNA-derived noncoding RNA targets and regulates the ribosome. *Mol. Cell* 54 147–155. 10.1016/j.molcel.2014.02.024 24685157 PMC3988847

[B53] Rice J. B. Vanderpool C. K. (2011). The small RNA SgrS controls sugar-phosphate accumulation by regulating multiple PTS genes. *Nucleic Acids Res.* 39 3806–3819. 10.1093/nar/gkq1219 21245045 PMC3089445

[B54] Romilly C. Deindl S. Wagner E. G. H. (2019). The ribosomal protein S1-dependent standby site in tisB mRNA consists of a single-stranded region and a 5′ structure element. *PNAS* 116 15901–15906. 10.1073/pnas.1904309116 31320593 PMC6690012

[B55] Sawyer A. J. Wesolowski D. Gandotra N. Stojadinovic A. Izadjoo M. Altman S. (2013). A peptide-morpholino oligomer conjugate targeting *Staphylococcus aureus* gyrA mRNA improves healing in an infected mouse cutaneous wound model. *Int J. Pharm.* 453 651–655. 10.1016/j.ijpharm.2013.05.041 23727592 PMC3756894

[B56] Schumann W. (2012). Thermosensor systems in eubacteria. *Adv. Exp. Med. Biol.* 739 1–16. 10.1007/978-1-4614-1704-0_1 22399392

[B57] Sharma C. M. Darfeuille F. Plantinga T. H. Vogel J. (2007). A small RNA regulates multiple ABC transporter mRNAs by targeting C/A-rich elements inside and upstream of ribosome-binding sites. *Genes Dev.* 21 2804–2817. 10.1101/gad.447207 17974919 PMC2045133

[B58] Shepherd J. Ibba M. (2015). Bacterial transfer RNAs. *FEMS Microbiol. Rev.* 39 280–300. 10.1093/femsre/fuv004 25796611 PMC4542688

[B59] Smirnov A. Förstner K. U. Holmqvist E. Otto A. Günster R. Becher D. (2016). Grad-seq guides the discovery of ProQ as a major small RNA-binding protein. *PNAS* 113 11591–11596. 10.1073/pnas.1609981113 27671629 PMC5068311

[B60] Sohmen D. Harms J. M. Schlünzen F. Wilson D. N. (2009). SnapShot: antibiotic inhibition of protein synthesis I. *Cell* 138 1248.e1–1248.e1. 10.1016/j.cell.2009.08.001 19766574

[B61] Sterk M. Romilly C. Wagner E. G. H. (2018). Unstructured 5’-tails act through ribosome standby to override inhibitory structure at ribosome binding sites. *Nucleic Acids Res.* 46 4188–4199. 10.1093/nar/gky073 29420821 PMC5934652

[B62] Studer S. M. Joseph S. (2006). Unfolding of mRNA secondary structure by the bacterial translation initiation complex. *Mol. Cell* 22 105–115. 10.1016/j.molcel.2006.02.014 16600874

[B63] Sully E. K. Geller B. L. Li L. Moody C. M. Bailey S. M. Moore A. L. (2017). Peptide-conjugated phosphorodiamidate morpholino oligomer (PPMO) restores carbapenem susceptibility to NDM-1-positive pathogens in vitro and in vivo. *J. Antimicrob. Chemother.* 72 782–790. 10.1093/jac/dkw476 27999041 PMC5890718

[B64] van de Waterbeemd M. Fort K. L. Boll D. Reinhardt-Szyba M. Routh A. Makarov A. (2017). High-fidelity mass analysis unveils heterogeneity in intact ribosomal particles. *Nat. Methods* 14 283–286. 10.1038/nmeth.4147 28114288

[B65] Ventola C. L. (2015). The antibiotic resistance crisis. *P T* 40 277–283. 25859123 PMC4378521

[B66] Villegas A. Kropinski A. M. (2008). An analysis of initiation codon utilization in the Domain Bacteria – concerns about the quality of bacterial genome annotation. *Microbiology* 154 2559–2661. 10.1099/mic.0.2008/021360-0 18757789

[B67] Vogel J. (2020). An RNA biology perspective on species-specific programmable RNA antibiotics. *Mol. Microbiol.* 113 550–559. 10.1111/mmi.14476 32185839

[B68] Weilbacher T. Suzuki K. Dubey A. K. Wang X. Gudapaty S. Morozov I. (2003). A novel sRNA component of the carbon storage regulatory system of *Escherichia coli*. *Mol. Microbiol.* 48 657–670. 10.1046/j.1365-2958.2003.03459.x 12694612

[B69] Yang L. Duff M. O. Graveley B. R. Carmichael G. G. Chen L.-L. (2011). Genomewide characterization of non-polyadenylated RNAs. *Genome Biol.* 12:R16. 10.1186/gb-2011-12-2-r16 21324177 PMC3188798

[B70] Yang Q. Figueroa-Bossi N. Bossi L. (2014). Translation enhancing ACA motifs and their silencing by a bacterial small regulatory RNA. *PLoS Genet.* 10:e1004026. 10.1371/journal.pgen.1004026 24391513 PMC3879156

[B71] Zhang J. Deutscher M. P. (1992). A uridine-rich sequence required for translation of prokaryotic mRNA. *Proc. Natl. Acad. Sci. U.S.A.* 89 2605–2609. 1372983 10.1073/pnas.89.7.2605 PMC48710

